# Subcritical Butane Extraction of Wheat Germ Oil and Its Deacidification by Molecular Distillation

**DOI:** 10.3390/molecules21121675

**Published:** 2016-12-07

**Authors:** Jinwei Li, Dewei Sun, Lige Qian, Yuanfa Liu

**Affiliations:** State Key Laboratory of Food Science and Technology, Collaborative Innovation Center of Food Safety and Quality Control in Jiangsu Province Jiangnan University, Wuxi 214122, China; jwli@jiangnan.edu.cn (J.L.); sundewei1912@163.com (D.S.); gzdeyouxiang@126.com (L.Q.)

**Keywords:** wheat germ oil, subcritical butane extraction, molecular distillation

## Abstract

Extraction and deacidification are important stages for wheat germ oil (WGO) production. Crude WGO was extracted using subcritical butane extraction (SBE) and compared with traditional solvent extraction (SE) and supercritical carbon dioxide extraction (SCE) based on the yield, chemical index and fatty acid profile. Furthermore, the effects of the molecular distillation temperature on the quality of WGO were also investigated in this study. Results indicated that WGO extracted by SBE has a higher yield of 9.10% and better quality; at the same time, its fatty acid composition has no significant difference compared with that of SE and SCE. The molecular distillation experiment showed that the acid value, peroxide value and *p*-anisidine value of WGO were reduced with the increase of the evaporation temperatures, and the contents of the active constituents of tocopherol, polyphenols and phytosterols are simultaneously decreased. Generally, the distillation temperature of 150 °C is an appropriate condition for WGO deacidification with the higher deacidification efficiency of 77.78% and the higher retention rate of active constituents.

## 1. Introduction

Wheat germ is a by-product of the wheat milling industry. It contains about 8%–14% oil and plays a crucial role in the food industry [1]. WGO is the most important source of tocopherol of all vegetable oils, up to about 2500 mg/kg [2], especially the content of α-tocopherol, which represents around 60% of the total tocopherol. WGO is also a rich source of unsaponifiable phytosterols, in particular sitosterol (60%–70%) and campesterol (20%–30%) [3], polycosanols (POC), thiamine, riboflavin and niacin. In addition, tetracosanol, hexa-cosanol, and octacosanol are the major POC components in all varieties of WGO [4,5,6]. Furthermore, WGO is high in human essential unsaturated fatty acids, including about 80% of linoleic (18:2) and linolenic (18:3) acids [4]. In recent years, WGO has been used as a functional additive in natural foods or cosmetic products. However, the presence of polyunsaturated fatty acids and bioactive compounds affects its shelf life because they are prone to oxidation and degradation [7].

The trends of natural and safe products have driven supercritical fluid technology to be a primary alternative to traditional solvent extraction (SE) for active ingredients of the food industry [8]. Although supercritical carbon dioxide extraction (SCE) has been widely used in the food industry [9], the higher processing pressure and operation cost have limited its application. Subcritical fluid extraction is performed at a lower temperature and pressure than those employed in SCE, which endow the subcritical fluid extraction with a higher application potential. Besides the mild temperature and pressure, the use of short-chain hydrocarbons, including propane or *n*-butane, allows the reduction of the extraction time because of their high density, diffusivity and low viscosity [10], while improving the quality of the oil including no use of toxic residual solvents, higher oxidation stability and reducing the degradation of the bioactive components [11,12]. Some results have been reported and have proved profitable and effective with the use of compressed n-butane [13] and some results have been reported with the use of compressed propane [10,14]. There are several works which compared the different extraction methods of SCE and SE and the extract composition. Fiori et al. investigated the mass transfer kinetics of SCE grape oil and compared the lipid profiles and tocol with that of SE [15]. However, there are few reports regarding the differences in the different extraction methods of SCE, subcritical fluid extraction and SE in aspects related to the yield, physicochemical properties, and fatty acid composition of the WGO.

Crude vegetable oils need to be refined to produce high quality and highly stable oils through elimination of undesirable compounds. A significant portion of the nutritional oil components is lost during conventional refining processes. Ghazani et al. investigated the minor constituents in canola oil processed by traditional and minimal refining methods and found that traditional neutralization removed 19.6% of the total tocopherol and 23.6% of the total free sterols [16]. Wang and Johnson examined the effect of conventional oil refining processes on WGO quality and found that deodorization conditions reduced the tocopherol content of WGO significantly [4].

Molecular distillation, a special high-vacuum distillation technology, is widely used in the oil industry because of its shorter residence time and good thermal stability, etc. It has been reported that molecular distillation exhibits outstanding performance for separation or purification of high-boiling-point mixtures such as octacosanol from transesterified rice bran wax [17], triacylglycerol from free fatty acids mixtures [18], carotenoids from palm oil [19] and the production of palm olein–enriched diacylglycerol [20]. Martinello et al. demonstrated that this method can obtain refined grape seed oil with a lower free fatty acid content and higher tocopherol recovery, using molecular distillation at the feed flow of 1.5 mL/min and the temperature of 220 °C [21]. Wu et al. optimized the molecular distillation conditions of crude low-calorie cocoa butter [22]. Solaesa et al. produced monoacylglycerols (MAGs)-enriched ω-3 polyunsaturated fatty acids using molecular distillation [23]. Plenty of studies pointed out that molecular distillation was an appropriate method to separate heat-sensitive and low-volatile compounds [24], and it provides a new and efficient approach to solving the rancidity of oils and fats in food. However, there are few works to be reported in the field of the molecular distillation–based deacidification of WGO.

The purpose of this research was to assess the feasibility of subcritical butane extraction (SBE) in WGO extraction and compare it to SCE and SE on the basis of the yield, quality and fatty acid composition of the crude WGO. Furthermore, the effects of the molecular distillation temperature on the acid value, peroxide value, anisidine value, tocopherol content, polyphenols content and phytosterols content of WGO were also investigated.

## 2. Results and Discussion

### 2.1. Extraction of Crude WGO and Its Quality

#### 2.1.1. Comparison of WGO Extracted by Three Methods by Yield and Quality

The yields of crude WGO obtained by SE, SCE and SBE are presented in Table 1. From Table 1, it can be noted that the extraction methods significantly affected the WGO yield (*p* < 0.05). The yields of the three extraction methods followed the order SE > SBE > SCE and were 9.24%, 9.10% and 8.78% (*w*/*w*) on a dry weight basis, respectively. However, the extraction yield of SBE was not significantly different compared to that of SE (*p* > 0.05). Compared to SCE, SBE positively affected the yield of WGO and increased it by 3.64%. These results agree with those obtained by Illés and co-workers [25], who also found that the solvating power of propane was much higher than that of CO_2_. Moreover, SBE performed under a lower pressure and temperature was beneficial to its application in the food industry, especially for the heat-sensitive components of the materials.

WG is rich in linoleic acid (LA) and lipoxygenase (LOX), which is prone to enzyme-induced oxidation. Therefore, the acid value, peroxide value, and *p*-anisidine value were used to assess the oxidized status of oils extracted by the three methods (Table 1). SE resulted in the highest acid value (AV), peroxide value (POV), and *p*-anisidine value (PAV) of WGO compared to SCE and SBE, which could be attributed to the peroxide formation in the extraction of WGO by SE due to its higher extraction temperature.

After the SBE, the solvent of the extract was exhausted for 45 min at the pressure of 0.1 Mpa and the temperature of 50 °C. The subcritical butane extraction process is safe and could provide a final product free of toxic solvent residues, which has been proved by many studies [11,12]. Therefore, SPE seems to be an appropriate method for WGO extraction from the aspect of oil yield and properties.

#### 2.1.2. Comparison of WGO Extracted by Three Methods by Fatty Acids Composition

The fatty acid composition of crude WGO by three extraction methods was analyzed by GC. As shown in Table 2, the WGO was rich in unsaturated fatty acids, which amounted to about 80% of the total fatty acids. The fatty acid compositions of WGO extracted by the three methods were similar and there was no significant difference (*p* > 0.05) between them. The major fatty acids of WGO were linoleic acid (C18:2), palmitic acid (C16:0), oleic acid (C18:1) and linolenic acid (C18:3), which accounted for 59.46%–59.55%, 16.90%–16.73%, 13.38%–13.56% and 6.64%–6.85%, respectively. Among the unsaturated fatty acids of WGO, polyunsaturated fatty acids accounted for more than 66%. Linoleic acid was the principal unsaturated fatty acid, followed by oleic acid, palmitic acid, stearic acid.

### 2.2. Molecular Distillation of WGO

#### 2.2.1. The Quality Index of WGO after Decolorization

The crude WGO was decolorized and analyzed; its quality index is shown in Table 3. Compared with those of the crude WGO, the acid value and *p*-anisidine value increased by 4.24% and 5.6% respectively, which was related to the higher decolorization temperature. However, the peroxide value decreased by 28.57% because of the stronger absorption activity of active clay to the hydroperoxide. The fatty acid composition of WGO had no significant difference after decolorization.

The refining process would significantly affect the nutritional components of oil and some natural nutrients after decolorization, which is shown in Table 4. After decolorization, the polyphenols content was 155.45 mg/kg, decreased by 17.79%. The total phytosterols content decreased by 4.99% after decolorization. The tocopherol content has no significant difference.

#### 2.2.2. Effect of Molecular Distillation Temperature on the Chemical Index of WGO

The principle of molecular distillation is to utilize the difference in the molecular mean free path to achieve the purpose of separation, wherein the molecular mean free path of motion is proportional to the temperature of the evaporation surface [26]. As can be seen from Table 5, when the evaporation temperature was in the range of 110~210 °C, the acid value of WGO extracted by SBE demonstrated a downward trend with the increase of the distillation temperature. The acid value of WGO decreased from 4.35 mg KOH/g to 1.18 mg KOH/g, which was related to the increase of the evaporation rate of the free fatty acids and other small molecules in the lighter phase and resulted in the decrease of the acid value in the heavier phase when the evaporation temperature increased from 110 to 210 °C.

The deacidification efficiency reached 77.78% at the distillation temperature of 150 °C. When the temperature was higher than 170 °C, the acid value of WGO declined slowly and the deacidification rate showed a slowly rising trend. When the evaporation temperature was between 110~130 °C, the peroxide value of WGO declined rapidly, from 2.25 mmol/kg down to 1.38 mmol/kg. It is due to the primary oxidation products of hydrogen peroxide being unstable and decomposition occurring in the face of a high temperature, which caused the decline of the peroxide value. On the other hand, there was no significant difference in the peroxide value of WGO (*p* > 0.05) with the increase of the evaporation temperature, which was related to the balance of the formation and decomposition of hydrogen peroxide. Due to the secondary small-molecule oxide being distilled off as a lighter phase, the anisidine value of WGO was similar to the peroxide value in the range of 110~150 °C; however, when the evaporation surface temperature rose to 170 °C, the anisidine value increased, possibly due to the natural micronutrients beginning to decompose under the higher temperature of the molecular distillation conditions, thereby generating small molecules, resulting in the increase of the anisidine value. Similar results were reported in the literature for other vegetable oils [21,27]. Moreover, as the higher temperature would cause the loss of natural micronutrients of the WGO, the distillation temperature of 150 °C is more appropriate in practical applications.

#### 2.2.3. Effect of Molecular Distillation Temperature on the Tocopherol of WGO

The effects of the molecular distillation temperature on the tocopherol of WGO are shown in Table 6. The distillation temperature significantly affected the contents of the total tocopherol, α-tocopherol, β-tocopherol and γ-tocopherol (*p* < 0.05), and their contents were negatively related to the temperature. When the distillation temperature increased from 110 °C to 150 °C, the total tocopherol, α-tocopherol, β-tocopherol and γ-tocopherol contents of the WGO decreased from 3709 to 3285.2 mg/kg, 2362.7 to 2246.2 mg/kg, 1007.7 to 874.3 mg/kg and 311.6 to 150.1 mg/kg, respectively. When the temperature was higher than 150 °C, the total tocopherol, α-tocopherol, β-tocopherol and γ-tocopherol contents of the WGO were reduced by a large margin. Compared to the WGO before molecular distillation, the total tocopherol decreased by 82.2% at 170 °C and by 89.7% at 210 °C, which also showed similar trends to α-tocopherol, β-tocopherol and γ-tocopherol. Two aspects contributed to this phenomenon: one was that when the temperature increased, the tocopherol moved into the lighter phase, which would cause a decrease in the heavier phase; the other was that at a high temperature condition, the tocopherol would be easily decomposed. Thus, the higher temperature condition is not beneficial to the retention of tocopherol in WGO and this result is supported by previous research [28].

#### 2.2.4. Effect of Molecular Distillation Temperature on the Content of Polyphenols of WGO

**Figure 1:**
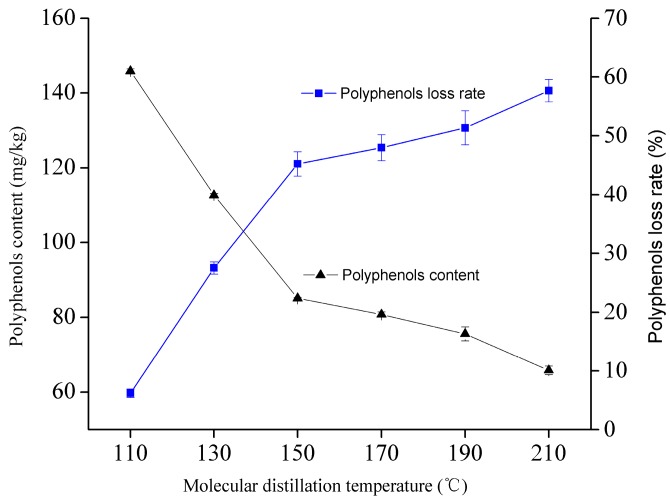
Effect of molecular distillation on the content of polyphenols of WGO.

The changes of the polyphenols of WGO after being treated by different molecular distillation temperatures are pictured in Figure 1. As can be seen, the distillation temperature significantly affected the content of the polyphenols (*p* < 0.05) and their contents decreased with the increase of the distillation temperature. When the temperature increased from 110 °C to 150 °C, the content of the polyphenols sharply decreased from 145.81 to 85.14 mg/kg. The polyphenols with relatively smaller molecular weights would enter the lighter phase at a higher temperature condition. However, when the experimental temperature was beyond 150 °C, the curve indicating the content of the polyphenols declined more slowly than the former, probably related to the higher vacuum condition, which could prevent the polyphenols from oxidation decomposition.

#### 2.2.5. Effect of Molecular Distillation Temperature on the Phytosterols of WGO

Generally, the contents of phytosterols decreased with the ascending temperature on the evaporating surface [29]. The results of these tests are shown in Table 7. The distillation temperature significantly affected the contents of the β-sitosterol, campesterol and stigmasterol (*p* < 0.05) and their contents were negatively related to the temperature. Among the phytosterols of WGO, β-Sitosterol was the most prominent, which accounted for 76%–84% of the total phytosterols, followed by campesterol and stigmasterol for all WGO samples distilled under different temperatures. For the WGO distilled under the temperature of 110–210 °C, the higher the temperature, the greater the loss rate of phytosterols in the heavier phase. The content of total phytosterols was 16.90 mg/g when the distillation temperature was 150 °C. When the temperature increased to 210 °C, the phytosterols decreased to 11.09 mg/g with a loss rate of 50.16%. Among the components of WGO, the loss of phytosterols was relatively lower, which was related to its higher boiling point and larger molecular weight making it difficult for them to transfer to a lighter phase when the distillation temperature was in the range of 110–150 °C. In contrast, with the rise of the distillation temperature, the phytosterols with lighter components would be more easily distilled, and their loss rate in the heavy phase became larger.

## 3. Materials and Methods

### 3.1. Chemicals

Wheat germ was obtained from WUDELI flour group co., LTD (Handan, Hebei, China). Mixed tocopherol standard (including α-, β-, γ-, δ-, 95%) (Roche, Basel, Switzerland), Gallic acid, isopropanol and dichloromethane were purchased from Sinopharm Chemical Reagent Co. (Shanghai, China).

### 3.2. Preparation of WGO

Untreated wheat germ was obtained from WUDELI flour group co., Ltd. (Handan, Hebei, China). After microwave enzyme inactivation at power of 600 W and time of 3 min, WG was extracted by SBE, then decolorized using activated clay of 3% at the temperature of 90 °C for 30 min. The decolorized WGO was disacidified by molecular distillation under the different temperature. The flow path of Preparation of WGO was shown as following:

Wheat germ → enzyme inactivation using microwave → extraction by SBE → decolorization → molecular distillation.

### 3.3. Extraction of WGO

WG after microwave enzyme inactivation was processed by subcritical butane extraction (SBE), and compared with the supercritical carbon dioxide extraction (SCE) and solvent extraction (SE), respectively according to the following methods.

#### 3.3.1. Extraction of WGO by SBE

**Figure 2:**
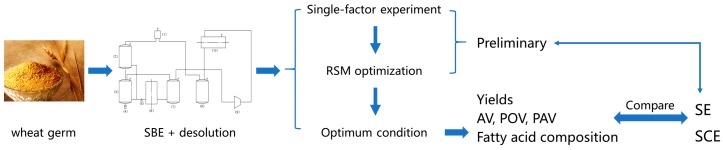
Flow diagram of subcritical fluid extraction.

SBE was performed using the apparatus (CBE-5L, Henan Yalinjie Biological Technology Co., Ltd., Anyang, China).The wheat germ (400 g) was loaded into the extractor, and the butane was compressed through a syringe-type pump, transferred to the extractor. The SBE was carried out at the pressure of 0.35 MPa and temperature of 35 °C for 1 h on the basis of the preliminary experiments. Butane was then exhausted at a temperature of 50 °C and the pressure of 0.1 MPa for 45 min. The obtained oil in the separators was collected, weighed and stored in sealed containers at 4 °C for further analysis. The flow diagram was shown in Figure 2.

#### 3.3.2. Extraction of WGO by SCE

All SCE trials were carried out in a HA121-50-01 SFE device (Hua’an Supercritical Fluid Extraction corp., Nantong, China) to get the WGO. The operating methodology was as follows: Approximately 400 g of wheat germ were loaded into the extraction vessel, carbon dioxide was pumped into the extractor until the desired extraction pressure values was reached. The wheat germ was extracted at pressure of 35 MPa and temperature of 45 °C for 2 h, and the liquid CO_2_ flow rate was set at 26 L/h. The operating conditions of the separators were set at 8 MPa and 30 °C. When the predetermined time was achieved, the obtained oil in the separators were collected and weighted and stored in sealed containers at 4 °C for further analysis.

#### 3.3.3. Extraction of WGO by SE

SE of wheat germ was performed with n-hexane as the extracting solvent. Then 400 g of wheat germ powders with 2 L hexane was placed into the beaker and stirred 5 h by digital-heating mantle at 50 °C and 400 rpm. After extraction, the hexane solution was filtered, and subsequently evaporated from the extracted oil in a rotary evaporator. The obtained oil was collected, weighed and stored in sealed containers at 4 °C for further analysis.

### 3.4. Molecular Distillation of WGO

The WGO were added to short-range molecular distillation equipment (KDL2UIC GmbH, Alzenau, Germany). The preheating and condensing temperature were set at 45 °C and 25 °C, respectively. The vacuum degree was adjusted to 0.01 mbar, and scraping film speed was set to 120 rpm. The feed rate was adjusted to 1 mL/min and distillation temperature was set at 110 °C, 130 °C, 150 °C, 170 °C, 190 °C, 210 °C, respectively.

### 3.5. Analytical Methods

The fatty acid composition of WGO was analyzed according to the IUPAC method 2.302 [30]. The tocopherol (α-, β-, γ-, and δ-isomers) contents of WGO were determined according to the method described by Liang, Yang and Ma [31]. Acid value (AV), peroxide value (POV), and *p*-anisidine value (PAV) were analyzed according to the method described by Li et al. [32]. The total phenolic content was determined by the Folin-Ciocaulteu method based on the method described by Li et al. [33]. Phytosterols were analyzed using a GC-MS (Thermo Electron, Waltham, MA, USA) according to the method described by Mitei et al. [34].

### 3.6. Statistical Analysis

Statistical analysis was carried out with SPSS 16.0 for Windows software (SPSS China, Shanghai, China). Graphics rendering was performed using Origin 8.6 software (OriginLab, Northampton, MA, USA). Differences were considered significant at *p* < 0.05.

## 4. Conclusions

SBE is an appropriate method for crude WGO extraction. When extracted by SBE at the pressure of 0.35 MPa and temperature of 35 °C for 1 h, the yield of WGO was 9.10%, which was higher than that of SCE and similar to that of SE. The AV, POV and PAV of WGO were 8.97 mg KOH/g, 4.69 meq/kg and 1.25, respectively. At the same time, the fatty acid composition of WGO extracted by SBE has no significant difference compared with that of SE and SCE.

Molecular distillation at 150 °C is an appropriate condition for WGO deacidification. At this condition, the deacidification efficiency of crude WGO was 77.78% and the contents of tocopherol, polyphenols and phytosterols were 3285.2 mg/kg, 85.14 mg/kg and 16.9 mg/g, respectively.

## Figures and Tables

**Table 1:** Extraction yield and chemical characterization of wheat germ oil (WGO) by three extraction methods.

Quality Index	Subcritical Butane Extraction (SBE)	Supercritical Carbon Dioxide Extraction (SCE)	Solvent Extraction (SE)
Oil yield (%, db.)	9.10 ± 0.08 ^a^	8.78 ± 0.08 ^b^	9.24 ± 0.11 ^a^
Acid value (AV, mg KOH/g)	8.97 ± 0.01 ^a^	10.51 ± 0.00 ^b^	11.19 ± 0.08 ^c^
Peroxide value (POV, meq/kg)	4.69 ± 0.58 ^a^	5.01 ± 0.01 ^a^	5.43 ± 0.59 ^a^
*p*-anisidine value(PAV)	1.25 ± 0.05 ^a^	1.23 ± 0.09 ^a^	1.65 ± 0.07 ^b^

^a^ Each value is expressed as mean ± standard deviation (*n* = 3). Means with different letters of ^a–c^ within a row are significantly different (*p* < 0.05) by Bonferroni *t*-test.

**Table 2:** Fatty acid composition of WGO obtained by three extraction methods (%).

FA	SBE	SCE	SE
C15:1	0.19	0.20	0.20
C16:0	16.83	16.90	16.73
C16:1	0.18	0.19	0.19
C18:0	0.56	0.54	0.56
C18:1	13.43	13.38	13.56
C18:2	59.52	59.55	59.46
C18:3	6.64	6.85	6.67
C20:1	1.36	1.38	1.38
C22:1	0.25	0.23	0.24
C24:1	0.24	0.22	0.21

**Table 3:** The chemical index and fatty acid composition of WGO after decolorization.

Quality Index	Decolorized WGO											
Chemical index	AV (mg KOH/g)				POV (meq/kg)				PAV			
	9.35 ± 0.088				3.35 ± 0.059				1.32 ± 0.045			
Fatty acid composition	C15:1	C16:0	C16:1	C18:0	C18:1	C18:2	C18:3	C20:0	C20:1	C21:0	C22:1	C24:1
	0.18	16.90	0.19	0.54	13.50	59.68	6.70	0.11	1.41	0.17	0.24	0.22

**Table 4:** Changes of natural nutrients in WGO after decolorization.

Natural Nutrients (mg/kg)	Crude WGO	After Decolorization
polyphenols	189.09 ± 0.19	155.45 ± 0.80
Tocopherol	3749.7 ± 17.7	3715.3 ± 13.5
α-tocopherol	2597.7 ± 20.4	2577.7 ± 4.2
β-tocopherol	1039.9 ± 2.3	1032.6 ± 12.2
γ-tocopherol	112.1 ± 5.1	105.0 ± 5.5
phytosterols	23,270 ± 590	22,110 ± 460
β-sitosterol	19,420 ± 550	18,760 ± 410
campesterol	2090 ± 50	2080 ± 60
stigmasterol	1760 ± 10	1270 ± 10

**Table 5:** Effect of molecular distillation on acid value, peroxide value and anisidine value of WGO.

Temperature	110 °C	130 °C	150 °C	170 °C	190 °C	210 °C
Acid value (mg KOH/g)	4.35 ± 0.21 ^e^	2.37 ± 0.11 ^d^	2.01 ± 0.07 ^c^	1.46 ± 0.14 ^b^	1.42 ± 0.06 ^a,b^	1.18 ± 0.07 ^a^
Peroxide value (mmol/kg)	2.25 ± 0.09 ^a^	1.38 ± 0.12 ^b^	1.25 ± 0.12 ^b^	1.29 ± 0.05 ^b^	1.16 ± 0.17 ^b^	1.26 ± 0.06 ^b^
*p*-anisidine	2.44 ± 0.14 ^c^	2.06 ± 0.04 ^a,b^	1.89 ± 0.12 ^a^	2.27 ± 0.13 ^b,c^	2.18 ± 0.16 ^a,b,c^	2.15 ± 0.11 ^a,b^

Each value is expressed as mean ± standard deviation (*n* = 3). ^a–e^ Means with different letters within a row are significantly different (*p* < 0.05) by Bonferroni *t*-test.

**Table 6:** Effect of molecular distillation on the content of tocopherol of WGO.

Tocopherol (mg/kg)	110 °C	130 °C	150 °C	170 °C	190 °C	210 °C
Total tocopherol	3709.4 ± 10.3 ^a^	3676.6 ± 18.4 ^a^	3285.2 ± 15.9 ^b^	661.7 ± 19.8 ^c^	485.9 ± 17.3 ^d^	383.5 ± 13.3 ^e^
α-tocopherol	2362.7 ± 15.6 ^a^	2422.7 ± 16.9 ^a^	2246.2 ± 16.9 ^b^	517.1 ± 19.6 ^c^	393.3 ± 21.3 ^d^	310.9 ± 19.7 ^e^
β-tocopherol	1007.7 ± 17.5 ^a^	994.4 ± 19.5 ^a^	874.3 ± 14.7 ^b^	121.0 ± 16.9 ^c^	75.1 ± 9.2 ^d^	58.7 ± 10.3 ^d^
γ-tocopherol	311.6 ± 11.2 ^a^	234.4 ± 3.6 ^b^	150.1 ± 2.4 ^c^	23.6 ± 2.1 ^d^	17.5 ± 1.2 ^d^	12.9 ± 0.6 ^d^

Each value is expressed as mean ± standard deviation (*n* = 3). ^a–e^ Means with different letters within a row are significantly different (*p* < 0.05) by Bonferroni *t*-test.

**Table 7:** Effect of molecular distillation on the content (mg/g) of phytosterols in WGO.

Phytosterols	110 °C	130 °C	150 °C	170 °C	190 °C	210 °C
β-sitosterol	17.92 ± 0.77 ^a^	15.68 ± 0.19 ^b^	14.05 ± 0.62 ^c^	12.25 ± 0.48 ^d^	11.34 ± 0.03 ^d,e^	10.27 ± 0.27 ^e^
campesterol	2.24 ± 0.19 ^a^	2.01 ± 0.04 ^a^	1.82 ± 0.38 ^a,b^	0.86 ± 0.15 ^b,c^	0.82 ± 0.17 ^b,c^	0.45 ± 0.60 ^c^
stigmasterol	1.29 ± 0.02 ^a^	1.23 ± 0.02 ^a^	1.03 ± 0.02 ^b^	0.83 ± 0.02 ^c^	0.70 ± 0.00 ^d^	0.37 ± 0.02 ^e^
Total phytosterols	21.45 ± 0.98 ^a^	18.92 ± 0.13 ^a,b^	16.90 ± 1.03 ^b^	13.94 ± 0.64 ^c^	12.86 ± 0.20 ^c,d^	11.09 ± 0.85 ^d^

Each value is expressed as mean ± standard deviation (*n* = 3). ^a–e^ Means with different letters within a row are significantly different (*p* < 0.05) by Bonferroni *t*-test.

## References

[1] Dunford N.T., Zhang M. (2003). Pressurized solvent extraction of wheat germ oil. Food Res. Int..

[2] Piras A., Rosa A., Falconieri D., Porcedda S., Dessi M.A., Marongiu B. (2009). Extraction of oil from wheat germ by supercritical CO_2_. Molecules.

[3] Michael E., Nurhan T.D. (2008). Bioactive components of commercial and supercritical carbon dioxide processed wheat germ oil. J. Am. Oil Chem. Soc..

[4] Wang T., Johnson L.A. (2001). Refining high-free fatty acid wheat germ oil. J. Am. Oil Chem. Soc..

[5] Irmak S., Dunford N.T., Milligan J. (2006). Policosanol contents of beeswax, sugar cane and wheat extracts. Food Chem..

[6] Irmak S., Dunford N.T. (2005). Policosanol contents and compositions of wheat varieties. J. Agric. Food Chem..

[7] Krings U., El-Saharty Y.S., El-Zeany B.A., Pabel B., Berger R.G. (2000). Antioxidant activity of extracts from roasted wheat germ. Food Chem..

[8] Ahangari B., Sargolzaei J. (2012). Extraction of pomegranate seed oil using subcritical propane and supercritical carbon dioxide. Theor. Found. Chem. Eng..

[9] Herrero M., Cifuentes A., Ibañez E. (2006). Sub- and supercritical fluid extraction of functional ingredients from different natural sources: Plants, food-by-products, algae and microalgae: A review. Food Chem..

[10] Zanqui A.B., Morais D.R., Silva C.M., Santos J.M., Gomes S.T.M., Visentainer J.V., Eberlin M.N., Cardozo-Filho L., Matsushita M. (2015). Subcritical extraction of flaxseed oil with *n*-propane: Composition and purity. Food Chem..

[11] Mariod A.A., Matthaus B., Ismail M. (2011). Comparison of supercritical fluid and hexane extraction methods in extracting kenaf (*Hibiscus cannabinus*) seed oil lipids. J. Am. Oil Chem. Soc..

[12] Passos C.P., Silva R.M., Silva F.A., Coimbra M.A., Silva C.M. (2010). Supercritical fluid extraction of grape seed (*Vitis vinifera* L.) oil. Effect of the operating conditions upon oil composition and antioxidant capacity. Chem. Eng. J..

[13] Tres M.V., Racoski J.C., Di Luccio M., Oliveira J.V., Treichel H., Oliveira D., Mazutti M.A. (2014). Separation of soybean oil/*n*-hexane and soybean oil/*n*-butane mixtures using ceramic membranes. Food Res. Int..

[14] Santos K.A., Bariccatti R.A., Cardozo-Filho L., Schneider R., Palu F., Silva C., Silva E.A. (2015). Extraction of crambe seed oil using subcritical propane: Kinetics, characterization and modeling. J. Supercrit. Fluids.

[15] Fiori L., Lavelli V., Duba K.S., Harsha P.S.C.S., Mohamed H.B., Guella G. (2014). Supercritical CO_2_ extraction of oil from seeds of six grape cultivars: Modeling of mass transfer kinetics and evaluation of lipid profiles and tocol contents. J. Supercrit. Fluids.

[16] Ghazani S.M., Garcia-Llatas G., Marangoni A.G. (2013). Minor Constituents in Canola Oil Processed by Traditional and Minimal Refining Methods. J. Am. Oil Chem. Soc..

[17] Chen F., Wang Z., Zhao G., Liao X., Cai T., Guo L., Hu X. (2007). Purification process of octacosanol extracts from rice bran wax by molecular distillation. J. Food Eng..

[18] Arzate-Martínez G., Jiménez-Gutiérrez A., García H.S. (2011). Experimental analysis and modeling of the separation of triacylglycerol and free fatty acid mixtures using molecular distillation. Ind. Eng. Chem. Res..

[19] Batistella C.B., Maciel M.W. (1998). Recovery of carotenoids from palm oil by molecular distillation. Comput. Chem. Eng..

[20] Yeoh C.M., Phuah E.T., Tang T.K., Siew W.L., Abdullah L.C., Choong T.S.Y. (2014). Molecular distillation and characterization of diacylglycerol-enriched palm olein. Eur. J. Lipid Sci. Technol..

[21] Martinello M., Hecker G., Del C.P.M. (2007). Grape seed oil deacidification by molecular distillation: Analysis of operative variables influence using the response surface methodology. J. Food Eng..

[22] Wu W., Wang C., Zheng J. (2012). Optimization of deacidification of low-calorie cocoa butter by molecular distillation. LWT-Food Sci. Technol..

[23] Solaesa Á.G., Sanz M.T., Falkeborg M., Beltrán S., Guo Z. (2016). Production and concentration of monoacylglycerols rich in omega-3 polyunsaturated fatty acids by enzymatic glycerolysis and molecular distillation. Food Chem..

[24] De Moraes E.B., Martins P.F., Batistella C.B., Alvarez M.E.T., Maciel Filho R., Maciel M.R.W. (2006). Molecular distillation. Appl. Biochem. Biotechnol..

[25] Illés V., Daood H.G., Perneczki S., Szokonya L., Then M. (2000). Extraction of coriander seed oil by CO_2_ and propane at superand subcritical conditions. J. Supercrit. Fluids.

[26] Zhang G., Liu J., Liu Y. (2013). Concentration of Omega-3 Polyunsaturated Fatty Acids from Oil of *Schizochytrium limacinum* by Molecular Distillation: Optimization of Technological Conditions. Ind. Eng. Chem. Res..

[27] Yang M., Liu C., Huang F., Zheng C., Zhou Q. (2011). Effect of dehulling treatment on the oxidative stability of cold-pressed low erucic acid rapeseed oil. J. Am. Oil Chem. Soc..

[28] Martinello M.A., Villegas M., Pramparo M.D.C. (2007). Retaining maximum antioxidative potency of wheat germ oil refined by molecular distillation. J. Agric. Food Chem..

[29] Eisenmenger M., Dunford N.T., Eller F., Taylor S., Martinez J. (2006). Pilot-scale supercritical carbon dioxide extraction and fractionation of wheat germ oil. J. Am. Oil Chem. Soc..

[30] Paquot C., Hauntfenne A. (1987). IUPAC Standard Methods for the Analysis of Oils, Fats and Derivatives.

[31] Liang S., Yang G., Ma Y. (2010). Chemical characteristics and fatty acid profile of foxtail millet bran oil. J. Am. Oil Chem. Soc..

[32] Li J.W., Cai W.C., Sun D.W., Liu Y.F. (2016). A Quick Method for Determining Total Polar Compounds of Frying Oils Using Electric Conductivity. Food Anal. Methods.

[33] Li J.W., Ding S.D., Ding X.L. (2005). Comparison of antioxidant capacities of extracts from five cultivars of Chinese jujube. Process Biochem..

[34] Mitei Y.C., Ngila J.C., Yeboah S.O., Wessjohann L., Schmidt J. (2009). Profiling of Phytosterols, Tocopherols and Tocotrienols in Selected Seed Oils from Botswana by GC-MS and HPLC. J. Am. Oil Chem. Soc..

